# Targeting of HER3 with Functional Cooperative miRNAs Enhances Therapeutic Activity in HER2-Overexpressing Breast Cancer Cells

**DOI:** 10.1186/s12575-018-0081-x

**Published:** 2018-08-08

**Authors:** Hui Lyu, Jingcao Huang, Zhimin He, Bolin Liu

**Affiliations:** 1 Department of Pathology, School of Medicine, University of Colorado Anschutz Medical Campus, MS-8104, 12801 E. 17th Ave, Aurora, CO 80045 USA; 2 Department of Hematology, Hematologic Research Laboratory, West China Hospital, Sichuan University, Chengdu, Sichuan China; 3 Cancer Research Institute and Affiliated Cancer Hospital, Guangzhou Medical University, Guangzhou, Guangdong China

**Keywords:** HER3, miRNA-replacement therapy, Functional cooperation, HER2, Breast cancer

## Abstract

**Background:**

The HER3 receptor functions as a major cause of drug resistance in cancer treatment. It is believed that therapeutic targeting of HER3 is required to improve patient outcomes. It is not clear whether a novel strategy with two functional cooperative miRNAs would effectively inhibit *erbB3* expression and potentiate the anti-proliferative/anti-survival effects of a HER2-targeted therapy (trastuzumab) and chemotherapy (paclitaxel) on HER2-overexpressing breast cancer cells.

**Results:**

Combination of miR-125a and miR-205, as compared to either miRNA alone, potently inhibited expression of HER3 in HER2-overexpressing breast cancer BT474 cells. Co-expression of the two miRNAs not only reduced the levels of phosphorylated erbB3 (P-erbB3), Akt (P-Akt), and Src (P-Src), it also inhibited cell proliferation and increased cells at G1 phase. A multi-miRNA lentiviral vector - the cluster of miR-125a and miR-205 - was constructed to simultaneously express the two miRNAs in HER2-overexpressing breast cancer cells. Concurrent expression of miR-125a and miR-205 via the miRNA cluster transfection significantly enhanced trastuzumab-mediated growth inhibition and cell cycle G1 arrest in BT474 cells and markedly increased paclitaxel-induced apoptosis in another HER2-overexpressing breast cancer cell line HCC1954.

**Conclusions:**

Here, we showed that functional cooperative miRNAs effectively suppressed *erbB3* expression. This novel approach targeting of HER3 was able to enhance the therapeutic efficacy of trastuzumab and paclitaxel against HER2-overexpressing breast cancer.

**Electronic supplementary material:**

The online version of this article (10.1186/s12575-018-0081-x) contains supplementary material, which is available to authorized users.

## Background

The HER receptor tyrosine kinase (RTK) family, including the epidermal growth factor receptor (EGFR, also known as HER1/erbB1), HER2 (erbB2/neu), HER3 (erbB3), and HER4 (erbB4), is arguably the most important receptor family in the context of development and tumorigenesis [1, 2]. The HER3 receptor has no or much lower intrinsic kinase activity [3, 4]. It frequently co-expresses and interacts with another RTK in cancer cells to activate oncogenic signaling, such as PI-3 K/Akt pathway, MEK/MAPK pathway, and Src kinase [3, 5, 6]. Recent studies have identified oncogenic *erbB3* gene mutations in colon and gastric cancers [7]; however, overexpression without gene alteration is still the major mechanism for HER3 to be associated with a worse survival in patients with a wide variety of solid tumors [8]. Indeed, elevated expression of HER3 has been shown to play a pivotal role in the development of HER2-overexpressing breast cancer [9, 10], castration-resistant prostate cancer (CRPC) [11], and ovarian cancer [12, 13]. Studies on the underlying mechanisms indicate that one of the major functions of HER3 signaling is to cause treatment failure in human cancers [14–16]. Especially in breast cancer, HER3 serves as a vital co-receptor of HER2, and its expression is a rate-limiting factor for HER2-induced breast cancer cell survival, proliferation, and progression [9, 10, 15]. We have shown that elevated expression of HER3 renders HER2-overexpressing breast cancer cells resistant to tamoxifen [17], HER2-targeted therapy (trastuzumab/Herceptin and lapatinib) [18, 19], and the chemotherapeutic agent paclitaxel [20]. It is believed that inhibition of HER3 signaling is required to overcome drug resistance and effectively treat the breast cancer patients with HER2-overexpressing tumors.

Although both HER2 and HER3 receptors play pivotal roles in breast tumorigenesis, only HER2-targeted therapy has been clinically used in the treatment of HER2-overexpressing breast cancer. To date, no HER3-targeted therapy has been approved for cancer treatment. Because of its lack of or low kinase activity [3, 4], targeting of HER3 with a blocking antibody (Ab) is the only strategy under preclinical studies [21, 22] and clinical investigations. We have shown that the fully human anti-HER3 monoclonal Ab MM-121 (Merrimack Pharmaceuticals, Inc., Cambridge, MA, USA), inhibiting ligand-dependent activation of HER3 [21, 22], is able to abrogate drug resistance and significantly enhance the antitumor activity of trastuzumab and paclitaxel against HER2-overexpressing breast cancer in vitro and in vivo [16, 23, 24]. Moreover, our recent findings support the notion that inhibition/downregulation of HER3 could be achieved by the class I HDAC inhibitor (HDACi) entinostat (or SNDX-275, MS-275) or the functional cooperative miRNAs [25–27]. While the mechanism of action of the miRNAs is different from the anti-HER3 blocking Abs, this novel approach aims to reduce the protein levels of HER3 rather than just inhibit its signaling, which may eliminate the chance for tumor cells to develop resistance after initial response.

In the current study, we have focused on studying the inhibitory effect of co-expression of miR-125a and miR-205 on *erbB3* expression. We have also investigated whether the newly identified miRNA-based strategy, targeting of *erbB3* with two functional cooperative miRNAs, can significantly enhance the anti-proliferative/anti-survival effects of trastuzumab and paclitaxel on HER2-overexpressing breast cancer cells.

## Methods

### Reagents and Antibodies

The restriction endonucleases XbaI and NotI and T4 DNA ligase were purchased from New England Biolabs, Inc. (Ipswich, MA, USA). Paclitaxel was from LC Laboratories (Woburn, MA, USA). Trastuzumab (Herceptin) and Ado-trastuzumab emtansine (T-DM1, also known as Kadcyla) were obtained from University of Colorado Hospital pharmacy. Antibodies used for western blot assays were as follows: erbB3 (Ab7) (LabVision Corp. Fremont, CA, USA); P-erbB3, caspase-8 (1C12), and caspase-3 (8G10), P-MAPK, MAPK, P-Akt (S473), Akt, P-Src(Y416), Src, PARP (Cell Signaling Technology, Inc., Beverly, MA, USA); E2F1, Cyclin D1, p27^kip1^ (Santa Cruz Biotechnology, Inc. Dallas, TX, USA); and β-actin (Sigma Co., St. Louis, MO, USA). All other reagents were purchased from Sigma Co. unless otherwise specified.

### Cells and Cell Culture

Human breast cancer cell lines SKBR3, BT474 and HCC1954 were obtained from the American Type Culture Collection (Manassas, VA, USA). The identity of all cell lines was confirmed with DNA profiling by our Cancer Center’s DNA Sequencing & Analysis Core facility. Cell lines were free of mycoplasma contamination, determined by the MycoAlert™ Mycoplasma Detection Kit (Lonza Group Ltd., Basel, Switzerland) every 3 months. All cell lines were maintained in DMEM/F-12 (1:1) medium containing 10% FBS, and cultured in a 37 °C humidified atmosphere containing 95% air and 5% CO2 and split twice a week.

### Lentiviral Vectors, Virus Production and Cell Infection

Lentiviral vector pCDH-CMV-MCS-EF1-Puro and pCDH-miR-125b were kindly provided by Dr. Paul Jedlicka (Department of Pathology, University of Colorado Anschutz Medical Campus, Aurora, CO, USA). Other miRNA expression vector for miR-125a or miR-205 was constructed by cloning the miRNA precursor into the multiple cloning site of pCDH-CMV-MCS-EF1-Puro. The primer sequences used to amplify the miRNA precursors by PCR were the following:

- miR-125a primers
- 5′---GCTCTAGATGCTGTGTCTCTGTGGCTTC---3′
- 5′---ATAAGAATGCGGCCGCGAGGCGCTCAGAGTAGGTTG---3′
- miR-205 primers
- 5′---GCTCTAGATATCTGGGTGGCTGTTTTGA---3′
- 5′---ATAAGAATGCGGCCGCGAGGCTTTTCAGTAGACAAGCAA--3′

Multi-miRNA expression Lentivector (cluster) was obtained from SBI system Bioscience LLC. (Palo Alto, CA, USA).

The lentiviral vector and lentivirus packaging plasmids pCMV-VSVG and pCMV-ΔA.9 were co-transfected into HEK293T cells with FuGene 6 (Roche Diagnostics Corp., Indianapolis, IN, USA). After 24 h, the culture media were replaced with fresh medium. The virus in conditioned medium were harvested in 3 consecutive days and filtered with low-protein binding filters (Millex-HV, 0.45-mm polyvinylidene difluoride; Millipore Corp., Billerica, MA, USA) before they were aliquoted and stored at − 80 °C freezer. Prior to infection, the lentivirus-containing media were thawed completely at room temperature, and mixed with a same amount of fresh medium containing polybrene (8 μg/ml). The culture media of the candidate breast cancer cells were then replaced with the lentivirus-containing media. After 24 h, the virus-infected cells were selected with puromycin (1 μg/ml) for 48 h, and then subjected to required experiments.

### Analysis of miRNA Expression

Total RNA, including small RNA, was extracted and purified using the miRNeasy Mini Kit (QIAGEN Inc., Valencia, CA, USA) following the manufacturer’s instructions. For miRNA analysis, TaqMan MicroRNA Reverse Transcription kit (Applied Biosystems Inc., Foster City, CA, USA) was first used to generate cDNA with the hairpin primers, which are specific to the mature miRNA and will not bind to the precursors. The expression levels of miR-125a, miR-125b, and miR-205 were then measured by real-time PCR using TaqMan MicroRNA Assays (assay ID: 002198, 000449, 000509, respectively; Applied Biosystems Inc.) according to the manufacturer’s protocol. RNU6B was used as an internal control to normalize all data using the TaqMan RNU6B Assay (assay ID: 001093; Applied Biosystems Inc.). RNU6B levels were unaffected by entinostat treatment. The relative miRNA levels were calculated using the comparative Ct method (ΔΔCt) as described previously [27].

### Western Blot Analysis

Protein expression levels were determined by western blot analysis as described previously [19, 28]. Equal amounts of total cell lysates were boiled in Laemmli SDS-sample buffer, resolved by SDS-PAGE, transferred to nitrocellulose membrane (Bio-Rad Laboratories, Hercules, CA, USA), and probed with the primary antibodies described in the figure legends. After the blots were incubated with horseradish peroxidase-labeled secondary antibody (Jackson Immuno Research Laboratories, West Grove, PA, USA), the signals were detected using the enhanced chemiluminescence reagents (GE Healthcare Bio-Sciences Corp., Piscataway, NJ, USA).

### Quantification of Apoptosis

An apoptotic ELISA kit (Roche Diagnostics Corp.) was used to quantitatively measure cytoplasmic histone-associated DNA fragments (mononucleosomes and oligonucleosomes) as previously reported [19, 23]. This enzyme immunoassay was performed according to the manufacturer’s instructions.

### Cell Proliferation Assay

The IncuCyte™ system (Essen BioScience, Inc., Ann Arbor, MI, USA) was used to kinetically monitor cell growth. It is an automated imaging platform providing real-time images and quantitative data generated throughout the entire cell culture process. Proliferation is successfully measured using an IncuCyte phase-only processing module as described previously [28]. Briefly, Cells were plated onto 96-well plates in 10%FBS DMEM/F12 medium or 0.5% FBS DMEM/F12 medium containing trastuzumab (20 μg/ml). The plate was placed into the IncuCyte™ system at 37 °C for 2–3 days. During this time of period, each well was repeatedly scanned and imaged at fixed time intervals (every 4 h). The data were analyzed by the IncuCyte software. Data reflects the means of three independent experiments.

### Flow Cytometric Analysis of Cell Cycle

Flow cytometric analyses were performed to define cell cycle distribution for treated and untreated cells [19, 23]. Briefly, cells grown in 100-mm culture dishes were harvested and fixed with 70% ethanol. Cells were then stained for total DNA content with a solution containing 50 μg /ml propidium iodide and 100 μg/ml RNase I in PBS for 30 min at 37 °C. Cell cycle distribution was analyzed at the Flow Cytometry Core Facility of University of Colorado Cancer Center with a FAC Scan flow cytometer (BD Biosciences, San Jose, CA, USA).

### Statistical Analysis

Statistical analyses of the experimental data were performed using a two-sided Student’s t test. Significance was set at the *P* < 0.05. All values are reported at the mean +/− SD from at least three independent experiments.

## Results

### Lentiviral Expression Vectors of miR-125a and miR-205 have been Established

**Fig. 1:**
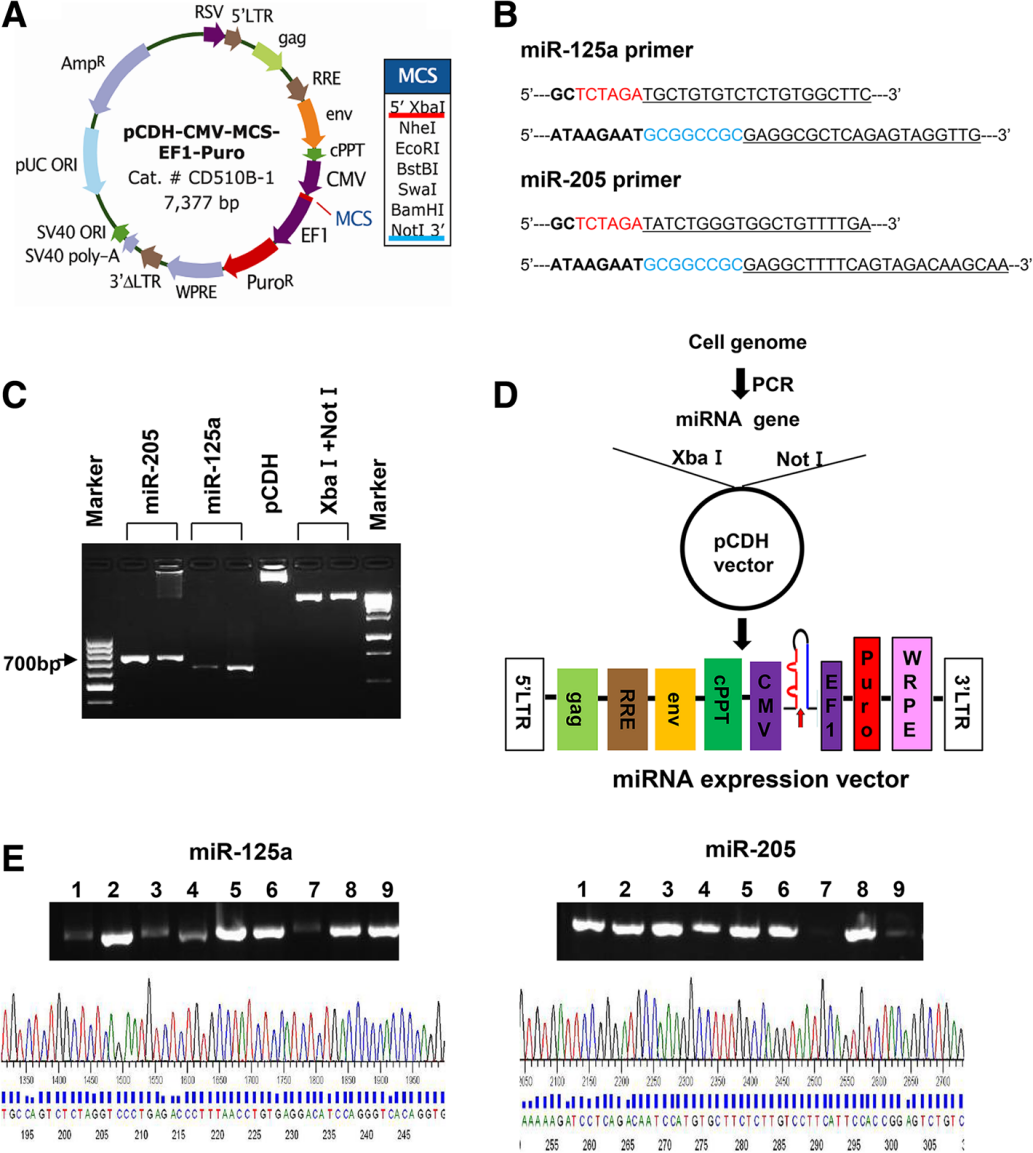
The lentiviral vector expressing miR-125a or miR-205 was constructed. **a** Map of the lentiviral vector pCDH-CMV-MCS-EF1-Puro. **b** Primers used in PCR assays to amplify the precursor of miR-125a or miR-205. The capital letters in red and blue indicate the digested sites of Xba I and Not I, respectively. **c** Electrophoretogram of the PCR products and the lentiviral vector digested with Xba I and Not I. **d** Strategy to construct the lentiviral vector with miRNA expression. **e** Results of PCR and DNA sequencing analysis of positive colonies

MiRNA-replacement therapy is actively explored as a novel strategy to treat human cancers [29–33]. While majority of the studies focus on the therapeutic potential of single miRNA, a new approach combining two miRNAs has been evaluated in lung cancer cells [34]. Our current studies aim to examine the hypothesis that functional cooperative miRNAs will effectively inhibit *erbB3* expression in HER2-overexpressing breast cancer cells. Thus, we decided to test our innovative idea with ectopic expression of miR-125a and/or miR-205. We utilized the lentiviral vector pCDH-CMV-MCS-EF1-Puro (pCDH) to construct the expression vectors of miR-125a and miR-205 (Fig. 1a). First, genome DNA extracted from SKBR3 cells was used as a template to amplify the precursors of miR-125a and miR-205 via the PCR primers shown in Fig. 1b. The PCR products and pCHD vector digested with the restriction endonucleases XbaI and NotI were then electrophoretically analyzed (Fig. 1c). After linked by a T4 DNA ligase, the resulting sub-clone vectors (Fig. 1d) were transformed into the DH5α bacteria. The plasmids obtained from the bacteria colonies (9 of each miRNA expression vector) were analyzed by PCR assays via the primers shown in Fig. 1b to evaluate the presence of the precursor of miR-125a or miR-205 (Fig. 1e top). Finally, one of the positive clones of miR-125a- or miR-205-expressing vector was further verified via DNA sequencing analysis (Fig. 1e bottom). Thus, the lentiviral expression vector of miR-125a (pCDH-miR-125a) or miR-205 (pCDH-miR-205) with accurate sequence has been established. The three miRNA expression vectors (pCDH-miR-125b obtained from Dr. Paul Jedlicka at our Department, pCDH-miR-125a, and pCDH-miR-205) were then used in the following experiments.

### Co-Expression of Two *erbB3*-Targeting miRNAs Shows Functional Cooperation and Potently Inhibits *erbB3* Expression in Human Breast Cancer Cells

**Fig. 2:**
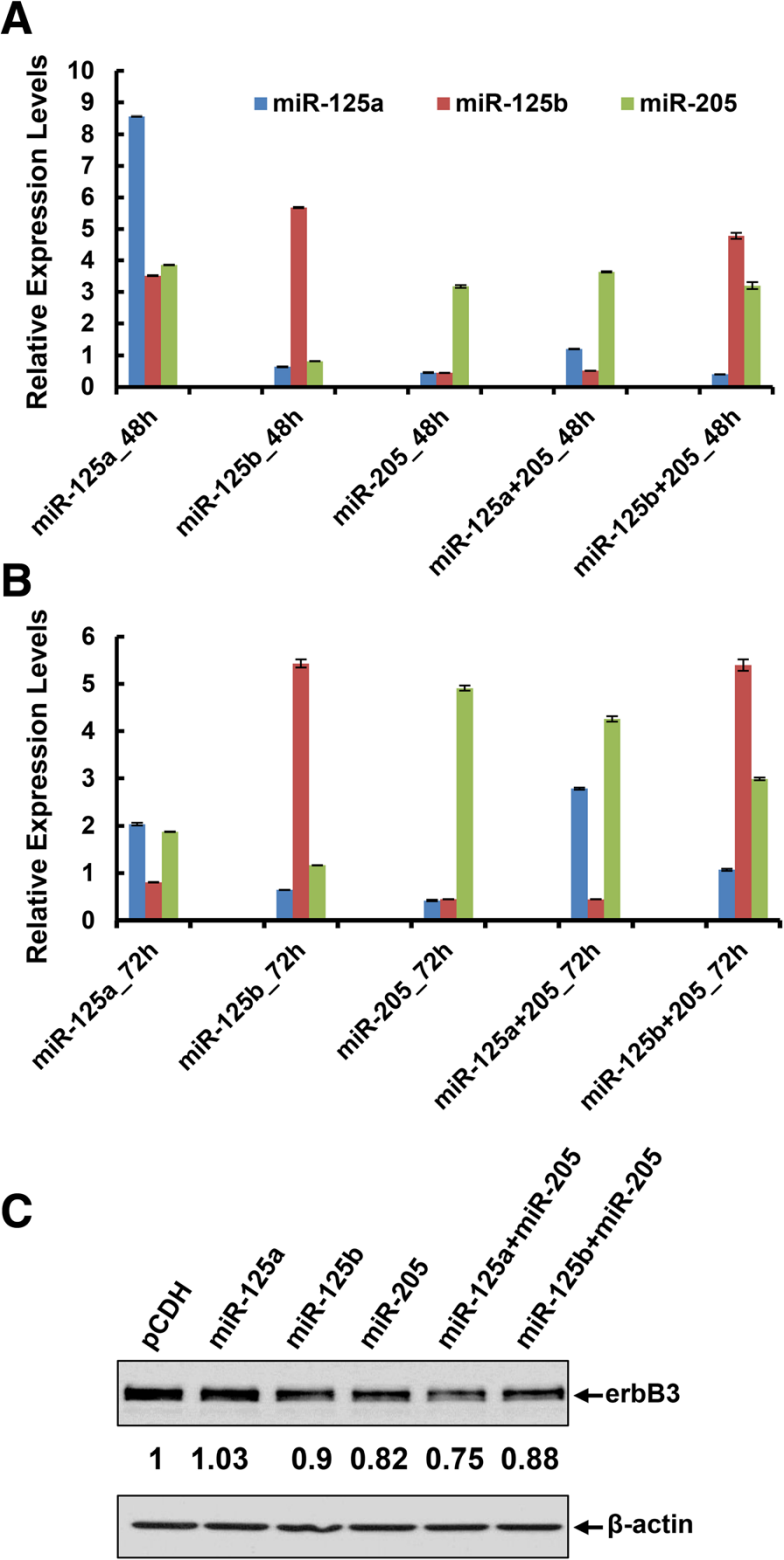
Ectopic expression of miR-125a, miR-125b, and/or miR-205 via transient transfection inhibited *erbB3* expression in HER2-overexpressing breast cancer cells. BT474 cells were infected with the lentivirus containing pCDH-miR-125a, pCDH-miR-125b, or pCDH-miR-205 alone or both pCDH-miR-125a and pCDH-miR-205 or both pCDH-miR-125b and pCDH-miR-205 for 48 h (**a**) or 72 h (**b**). The expression levels of miR-125a, miR-125b, and miR-205 were measured by qRT-PCR using TaqMan miRNA assays. All results were normalized with the internal control RNU6B. Bars, SD. Data shows the representative of three independent experiments. **c** The expression levels of HER3 (erbB3) were detected by western blot analysis upon lentiviral infection for 72 h. β-actin was used as a loading control

The human HER2-overexpressing breast cancer BT474 cells were seeded onto 6-well plate for overnight. The cells were then infected with the lentivirus containing pCDH-miR-125a, pCDH-miR-125b, or pCDH-miR-205 alone or both pCDH-miR-125a and pCDH-miR-205 or both pCDH-miR-125b and pCDH-miR-205 for 48 h (Fig. 2a) or 72 h (Fig. 2b). Taqman real-time PCR analyses revealed that the expression of each miRNA was specifically increased about 2–8 folds as compared to the empty vector (pCDH) control. Although all three miRNAs (miR-125a, miR-125b, and miR-205) have been shown to directly target the 3’UTR of *erbB3* mRNA [35, 36], a clear reduction of HER3 protein levels was only observed with ectopic expression of miR-205, but not miR-125a or miR-125b. Interestingly, the combinations of miR-125a and miR-205 or miR-125b and miR-205 were able to profoundly reduce HER3 levels (Fig. 2c). It appeared that co-expression of miR-125a and miR-205 exhibited a more potent inhibitory effect on HER3. Collectively, these data suggested that the expression of miR-125a, miR-125b, and/or miR-205 could be specifically increased in breast cancer cells; and two miRNAs, especially miR-125a and miR-205 showed functional cooperation to suppress *erbB3* expression in HER2-overexpressing breast cancer cells.

### Concurrent Expression of a Multi-miRNA Cluster Targeting of *erbB3* Significantly Inhibits Proliferation of HER2-Overexpressing Breast Cancer Cells

**Fig. 3:**
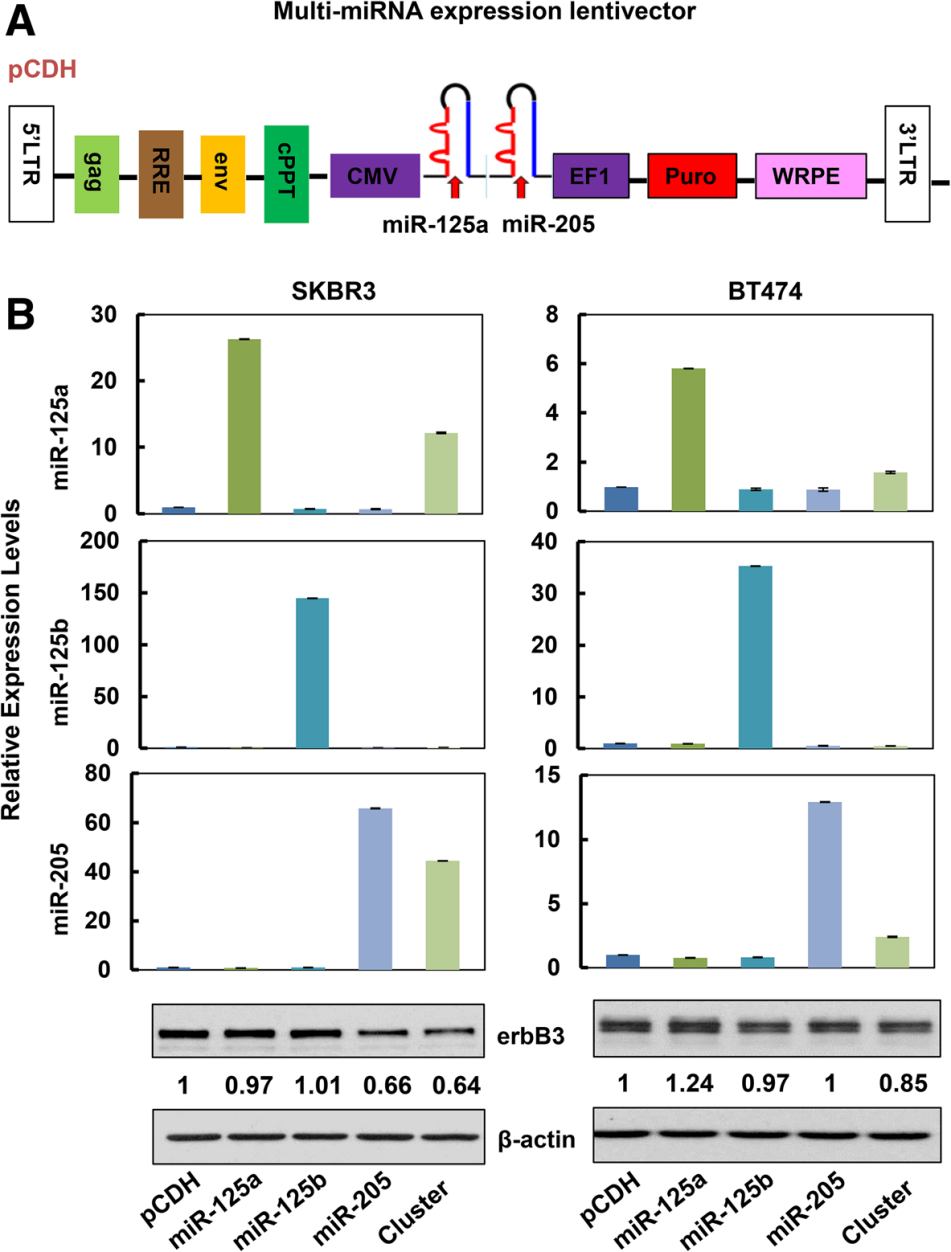
Co-expression of miR-125a and miR-205 via transient transfection with the multi-miRNA expression vector (cluster) exhibited potent activity to inhibit *erbB3* expression in HER2-overexpressing breast cancer cells. **a** Map of the multi-miRNA lentivector expression vector (cluster). The precursors of miR-125a and miR-205 were linked together and driven by the same CMV promoter. **b** SKBR3 cells and BT474 cells were infected with the lentivirus containing pCDH-miR-125a, pCDH-miR-125b, or pCDH-miR-205 alone or the pCDH-miR-125a-miR-205 (cluster) for 48 h. The expression levels of miR-125a, miR-125b, and miR-205 were measured by qRT-PCR using TaqMan miRNA assays. All results were normalized with the internal control RNU6B. Bars, SD. Data shows the representative of three independent experiments. The expression levels of HER3 (erbB3) were detected by western blot analysis upon lentiviral infection for 72 h. β-actin was used as a loading control

We hypothesized that functional cooperative miRNAs targeting of *erbB3* would be a powerful strategy to develop a miRNA-replacement therapy. However, delivering two distinct miRNAs at same time into a cancer cell may not be efficient and convenient. To better address our hypothesis, we then designed a multi-miRNA lentiviral vector simultaneously expressing both miR-125a and miR-205 and constructed the expression vector (Fig. 3a), which was designated as “miRNA cluster” in the following studies. Two HER2-overexpressing breast cancer cell lines SKBR3 and BT474 were used to test the efficacy of the miRNA cluster. The expression levels of both miR-125a and miR-205 were increased upon infection with the lentivirus containing the cluster (Fig. 3b bar graphs). Although the fold induction of miR-125a or miR-205 in cluster transfection was much less than that in single miRNA transfection, the miRNA cluster exhibited a much more potent activity than any one miRNA to downregulate HER3 in both SKBR3 and BT474 cells (Fig. 3b western blots). The data supported that two miRNAs expressed simultaneously via one vector, even at a very low expression level, could clearly inhibit *erbB3* expression.

**Fig. 4:**
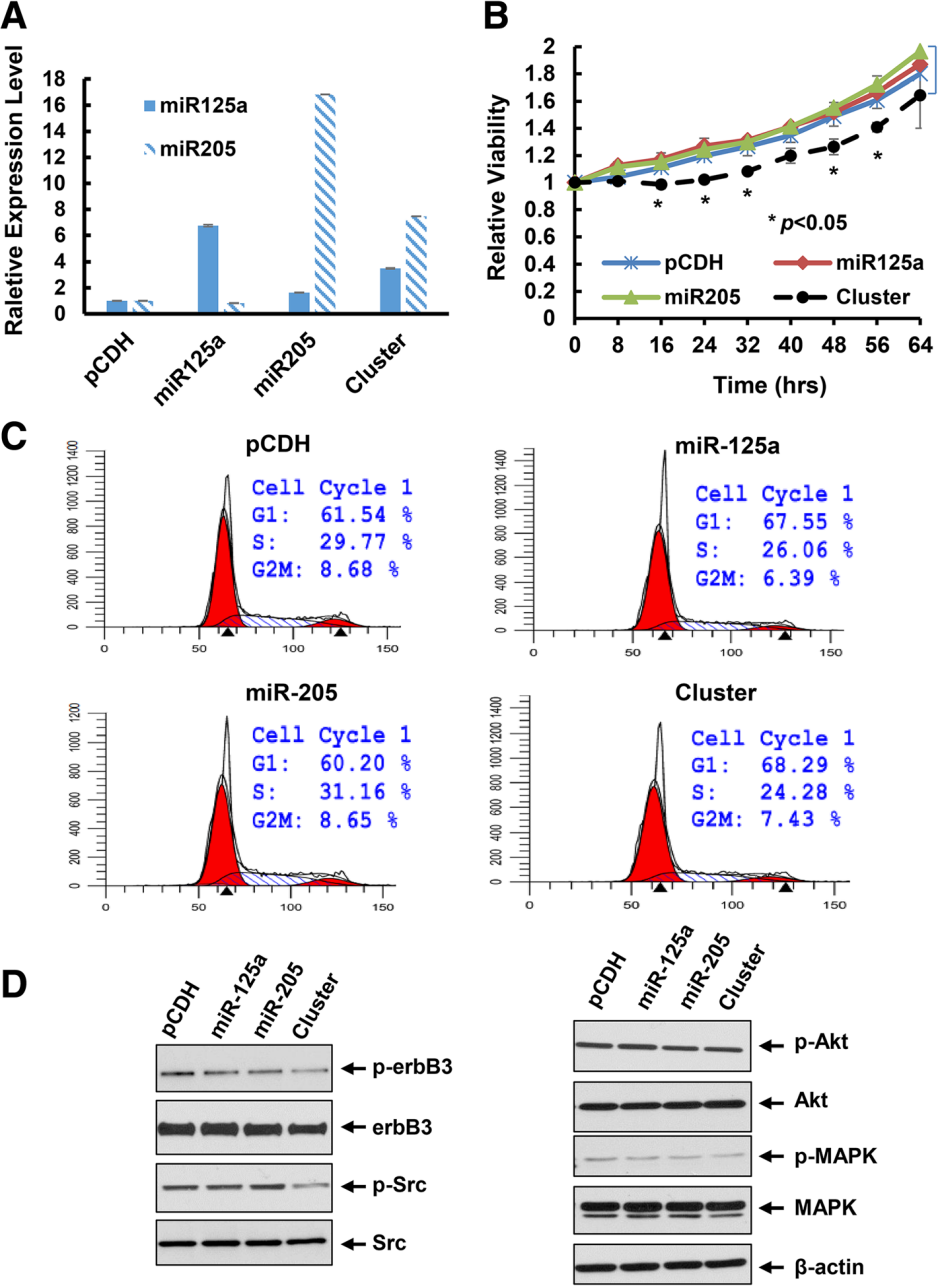
Concurrent expression of miR-125a and miR-205 via transfection with the miRNA cluster significantly inhibited proliferation, induced cell cycle G1 arrest, and inactivated HER3 signaling in BT474 cells. **a** The stable sub-clones of BT474 cells transfected with empty vector (pCDH), pCDH-miR-125a, or pCDH-miR-205 alone or pCDH-miR-125a-miR-205 (cluster) were selected. The relative expression levels of miR-125a and miR-205 were measured by qRT-PCR using TaqMan miRNA assays. In the cluster cell clone, both miR-125a and miR-205 were upregulated. **b** Proliferation of each cell clone was examined via analysis of the IncuCyte system. Bars, SD. Data shows the representative of three independent experiments. **c** Cell cycle progression was analyzed by flow cytometry. **d** The stable sub-clones were collected and subjected to western blot analyses with the specific antibodies directed against p-erbB3, HER3 (erbB3), p-Src, Src, p-Akt, Akt, p-MAPK, MAPK, or β-actin

We next examined the effects of ectopic expression of miR-125a, miR-205, or the cluster on proliferation of HER2-overexpressing breast cancer cells. The empty vector pCDH was used as a negative control. Upon infection of BT474 cells with the lentivirus containing pCDH-miR-125a, pCDH-miR-205, or pCDH-miRNA cluster, we obtained a number of cell clones from each lentiviral infection group upon puromycin selection. To avoid any potential artificial effects caused by induction of hundred- or thousand-fold of the miRNAs, we on purposely selected the cell clones that had increased expression levels of miR-125 and/or miR-205 within 10–20 folds as compared the empty vector control. Real-time PCR analyses showed that the expression of miR-125a was increased about 6-fold and the expression of miR-205 was enhanced about 16-fold in single miRNA transfected cell clones. In the cluster transfection, the expression of miR-125a and miR-205 was increased approximately 3- and 8-fold, respectively (Fig. 4a). These particular cell clones were designed as pCDH, miR125a, miR205, or cluster in the following experiments. While the miR125a and miR205 cell clones grew at a similar rate as the control (pCDH) cell clone, the cluster cell clone experienced a significant growth inhibition (Fig. 4b), although the fold induction of miR-125a and miR-205 was much less in the cluster than that in single miRNA transfected clones (Fig. 4a). Cell cycle analysis revealed that the cluster cell clone had increased percentage of cells at G1 phase and reduced cells at S phase (Fig. 4c). This might shed a light on the mechanism of growth inhibition-induced by the miRNA cluster. Furthermore, the expression of erbB3 and its phosphorylation (p-erbB3) as well as the downstream signaling kinases - phosphorylated Akt (p-Akt), MAPK (p-MAPK) and Src (p-Src) were also decreased in the cluster clone (Fig. 4d). Collectively, our data demonstrated that concomitant expression of the miRNA cluster targeting of *erbB3* significantly inhibited proliferation of HER2-overexpressing breast cancer cells, likely via cell cycle G1 arrest as well as inactivation of the HER3 signaling pathways.

### The miRNA Cluster Targeting of *erbB3* Enhances Anti-Proliferative/Anti-Survival Effects of Trastuzumab and Paclitaxel on HER2-Overexpressing Breast Cancer Cells

**Fig. 5:**
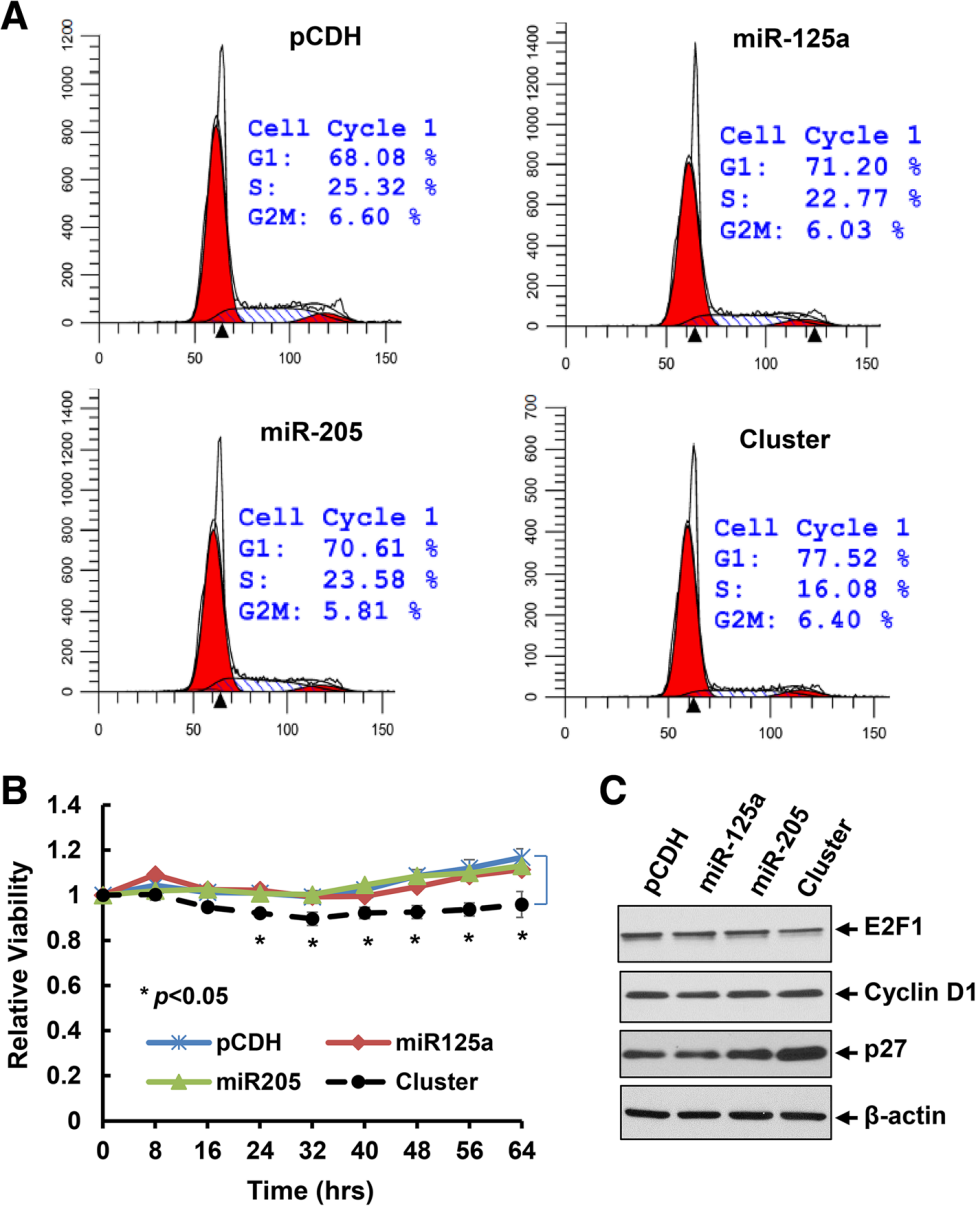
Co-expression of miR-125a and miR-205 via cluster transfection profoundly enhanced trastuzumab-induced growth inhibition and cell cycle G1 arrest in BT474 cells. The stable sub-clones of BT474 cells treated with trastuzumab (20 μg/ml) for 48 h were collected and subjected to cell cycle analysis by flow cytometry (**a**) and cell proliferation (the IncuCyte system) (**b)**. **c** The stable sub-clones of BT474 cells treated with trastuzumab (20 μg/ml) for 24 h were collected and subjected to western blot analysis with the specific antibodies directed against E2F1, Cyclin D1, p27^kip1^ (p27), or β-actin

**Fig. 6:**
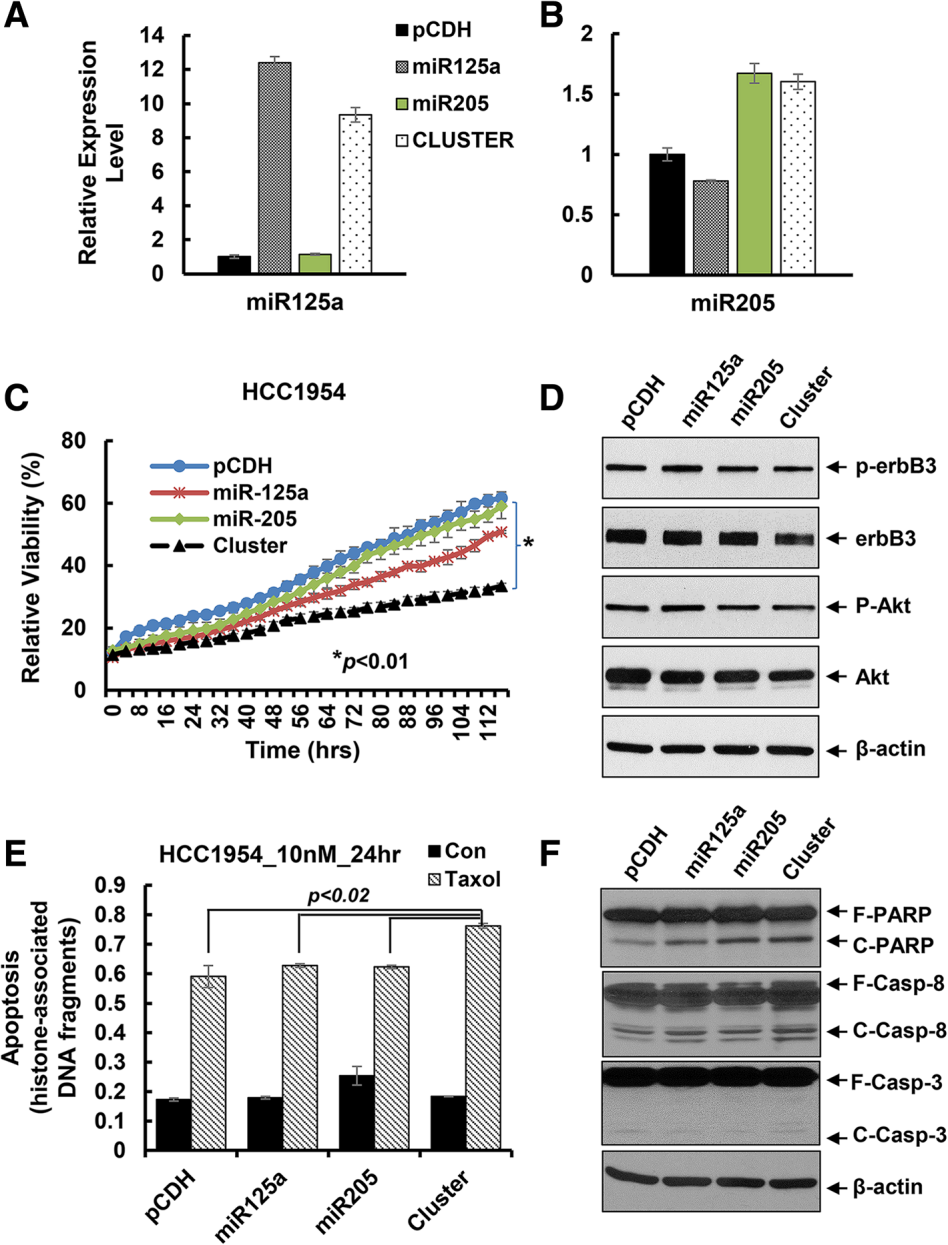
The miRNA cluster transfection significantly increased paclitaxel-mediated cytotoxicity effects on HCC1954 breast cancer cells. **a** & **b** The stable sub-clones of HCC1954 cells transfected with empty vector (pCDH), pCDH-miR-125a, or pCDH-miR-205 alone or pCDH-miR-125a-miR-205 (cluster) were selected. The relative expression levels of miR-125a and miR-205 were measured by qRT-PCR using TaqMan miRNA assays. **c** Proliferation of the sub-clones was measured by analysis of the IncuCyte system. **d** The stable sub-clones were collected and subjected to western blot analyses of p-erbB3, HER3 (erbB3), p-Akt, Akt, or β-actin. **e** & **f** Each sub-clone treated with paclitaxel (10 nmol/L) for 24 h were collected and subjected to apoptotic-ELISA (**e**) or western blot analyses with specific antibodies directed against (F-PARP, full length PARP; C-PARP, cleaved PARP), caspase-8 (F-Casp-8, full length caspase-8; C-Casp-8, cleaved caspase-8), caspase-3 (F-Casp-3, full length caspase-3; C-Casp-3, cleaved caspase-3), or β-actin (**f**). Bars, SD. Data shows the representative of three independent experiments

Our recent studies showed that activation of HER3 signaling and/or elevated expression of HER3 resulted in therapeutic resistance to trastuzumab and paclitaxel in HER2-overexpressing breast cancer cells [18, 20]. Thus, we first investigated whether inhibition of *erbB3* expression with the miRNA cluster could enhance the anti-proliferative effects of trastuzumab on HER2-overexpressing breast cancer cells. The pCDH, miR125a, miR205, and cluster cell clones described above were treated with trastuzumab (20 μg/ml) for 24 h. Cell cycle analysis showed a remarkable G1 arrest and S phase reduction in the cluster clone than the single miRNA clone or the vector control (Fig. 5a). Cell proliferation was more significantly inhibited in the cluster clone as compared to that in miR125a or miR205 clone or the vector control clone (Fig. 5b). Western blots evaluating the molecular markers critical for G1-S transition found a clear reduction of E2F1 and a striking upregulation of p27^kip1^ in the cluster clone-treated by trastuzumab (Fig. 5c). Similar results were also obtained with the BT474 cell clones treated by another HER2-targeted therapy, T-DM1, i.e. the cluster cell clone compared with the single miRNA clone or the vector control was more sensitive to T-DM1-induced growth inhibition (Additional file 1: Figure S1). We next tested the antitumor activity of paclitaxel using another HER2-overexpressing breast cancer cell line HCC1954 upon infection with the lentivirus containing pCDH-miR-125a, pCDH-miR-205, or pCDH-miRNA cluster. Same process was performed to select proper cell clones of each miRNA transfection. The expression levels of miR-125a and miR-205 were upregulated by approximately 12-fold and 1.6-fold, respectively in the single miRNA transfected clones, whereas about 9-fold and 1.5-fold increase of miR-125a and miR-205, respectively was observed in the cluster-transfected clone (Fig. 6a & b). The proliferation of HCC1954 cells was significantly inhibited in the cluster cell clone as compared to the cell clones with single miRNA transfection or vector control (Fig. 6c), which was in agreement with our experimental data obtained with BT474 cells (Fig. 4b). The expression of HER3, p-HER3, and p-Akt was also decreased in the cluster cell clone, consistent with our observation in BT474 cells (Fig. 4d). The HCC1954 cell clones were then challenged by paclitaxel (10 nmol/L) for 24 h. An apoptotic ELISA revealed significantly more cells undergoing apoptosis in the cluster cell clone (Fig. 6e). Western blot assays also detected more PARP cleavage, a hallmark of apoptosis, and increased cleaved caspase-8 and caspase-3 (Fig. 6f). Taken together, our data indicated that the miRNA cluster (miR-125a and miR-205) targeting of *erbB3* significantly enhanced anti-proliferative/anti-survival effects of trastuzumab and paclitaxel on HER2-overexpressing breast cancer cells.

## Discussion

Mounting evidence implicates that the major function of the HER3 receptor in human cancers is to cause treatment failure [14–16]. Effective inhibition of HER3 is thought to be required to overcome drug resistance, enhance therapeutic efficacy, and improve outcomes of cancer patients. Among the anti-HER3 monoclonal Abs actively under preclinical investigations and clinical testing [21, 37–39], two of them - MM-121 (or seribantumab, Merrimack Pharmaceuticals, Inc.) and patritumab (Daiichi-Sankyo Co. Ltd., Tokyo, Japan) - have shown encouraging clinic benefits in patients with non-small cell lung cancer [40, 41]. Thus, it is not surprising that the U.S. Food and Drug Administration (FDA) has recently (10/30/2017) granted orphan drug designation to MM-121 for the treatment of heregulin, which is the ligand for HER3, positive non-small cell lung cancer (http://investors.merrimack.com/node/11346). In an attempt to identify novel therapeutics and approaches inhibiting HER3 receptor, we discovered that the HDACi entinostat selectively inhibited HER3 expression in HER2-overexpressing breast cancer cells [25]. Further studies revealed that entinostat induced expression of miR-125a, miR-125b, and miR-205, all of which were reported to directly target the 3’UTR of *erbB3* mRNA [35, 36], and the three miRNAs acted in concert to inhibit HER3 protein translation in HER2-overexpressing breast cancer cells [27]. These exciting data support the hypothesis that effective targeting of HER3 could also be achieved by the HDACi entinostat or functional cooperative miR-125a, miR-125b, and miR-205. Because this novel approach aims to reduce HER3 protein levels, not just inhibits HER3 signaling, it has potential to eliminate the chance for tumor cells to develop resistance after initial response. To test this hypothesis, we decided to examine our innovative idea with miR-125a and miR-205, and to determine whether co-expression of the two miRNAs will exert functional cooperation to inhibit *erbB3* expression in HER2-overexpressing breast cancer cells. We did not chose miR-125b in our studies, because miR-125b has been shown to behave as an oncomir under certain circumstances [42, 43]. In contrast, both miR-125a and miR-205 are consistently found to act as tumor suppressors in breast cancers [44, 45]. Indeed, our data showed that co-expression of miR-125a and miR-205 was more potent than either miRNA alone to inhibit *erbB3* expression, decrease the levels of p-HER3, p-Src, p-Akt, and E2F1, and induce expression of p27^kip1^ in HER2-overexpressing breast cancer cells. It is worth mentioning that entinostat seems to be more powerful than our approach to repress erbB3 expression. However, the HDACi clearly shows additional side effects. In our study, since we on purposely increased expression of each miRNA at a limited amount, each single miRNA did not show much effect on erbB3 expression. However, the combination of two miRNAs exhibited a cooperative effect on inhibiting erbB3. We believe this innovative approach would be superior to entinostat in reducing the off-target effects.

MiRNA-based therapy is actively explored as a new strategy to treat human diseases, including cancer [46]. A locked nucleic acid (LNA)-modified miR-122 antagonist, named miravirsen/ SPC3649 (Santaris Pharma A/S, Copenhagen, Denmark), is the first miRNA reached clinical testing for the treatment of hepatitis C. It is currently under phase II clinical trial in patients with hepatitis C (http://clinicaltrials.gov/ct2/results?term=mir122&Search=Search). Because of their regulatory potential on the entire signaling networks within the cells and involvement in cancer development and progression, miRNAs have also emerged as promising molecular targets for the treatment of human cancers [29, 30, 33, 47]. Recent studies in this area drive the development of miRNAs as cancer therapeutics moving quickly from bench to clinical application [46, 48, 49]. In May 2013, a clinical trial using miR-34 mimics (trade name: MRX34, Mirna Therapeutics, Austin, TX) as a replacement therapy (ClinicalTrials.gov Identifier: NCT01829971) was initiated. This was the first and only miRNA replacement therapy carried out under clinical trials in multiple human cancers up until now [32, 50]. Unfortunately, the company (Mirna Therapeutics, Inc.) voluntarily halted patient enrollment and closed the phase 1 study of MRX34 on September 20, 2016, following multiple immune-related severe adverse events (SAE) observed in patients dosed with MRX34 over the course of the trial. It was hoped that further analysis of its full preclinical and clinical data set would provide useful information on the future development of MRX34 as a cancer therapeutics (https://www.bizjournals.com/austin/news/2016/09/21/austin-drug-company-halts-clinical-studies-after.html).

While majority of the basic research and clinical evaluations focusing on the therapeutic potential of one miRNA, a new approach combining two miRNAs has just been studied in lung cancer cells [34]. Considering this innovative idea, our research focused on determining the functional cooperation of miR-125a and miR-205 in suppressing *erbB3* expression. We on purposely selected the cell clones with an increased expression of miR-125a and/or miR-205 around 10-fold as compared to the cells transfected with the empty vector control (Figs. 2, 3, 4 and 6). With this limited elevation of miRNA expression levels, single miRNA (miR-125a or miR-205) enhancement had minor effect on *erbB3* expression, whereas co-expression of the two miRNAs significantly inhibited *erbB3* (Figs. 2c, 3b, & 4d). This approach would reduce the non-specific targeting of each miRNA, supporting the notion that “sister” miRNAs, which have common targets [51], may exert synergistic activity to only repress their common targets. Moreover, ectopic expression of both miR-125a and miR-205, as compared to either miRNA alone, not only inhibited proliferation, it also significantly enhanced trastuzumab-mediated growth inhibition and cell cycle G1 arrest and paclitaxel-induced apoptosis in HER2-overexpressing breast cancer cells. Thus, concurrent expression of “sister” miRNAs may be a more promising miRNA-replacement therapy for cancer treatment.

## Conclusions

In the current study, we demonstrate that co-expression of miR-125a and miR-205 is more potent than that of single miRNA to inhibit cell proliferation and enhance trastuzumab- and paclitaxel-mediated anti-proliferative/anti-survival effects on HER2-overexpressing breast cancer cells. Our data support further investigations developing the miRNA (miR-125a/miR-205) cluster as a novel, effective HER3-targeted therapy to enhance the efficacy of trastuzumab and paclitaxel against HER2-overexpressing breast cancer.

## Additional file

Additional file 1: **Figure S1.** Transfection with the multi-miRNA (cluster) lentiviral vector significantly enhanced T-DM1-induced growth inhibitory effects on BT474 cells. The stable clones of BT474 cells transfected with empty vector (pCDH), pCDH-miR-125a, or pCDH-miR-205 alone or pCDH-miR-125a-miR-205 (cluster) were seeded onto 96-well plates. After 24 hrs, the 96-well plates were then placed into the IncuCyte system to measure cell proliferation in a real time. Data show growth curves of the clones in comparison with their responses to the treatment of T-DM1 (10 μg/ml) in the indicated time period. (TIF 2615 kb)

## Abbreviations

Ab: Antibody
CPRC: Castration-resistant prostate cancer
EGFR: Epidermal growth factor receptor
ELISA: Enzyme-linked immunosorbent assay
FDA: Food and Drug Administration
HDAC: Histone deacetylase
HDACi: HDAC inhibitor
HER: Human epidermal growth factor receptor
LNA: Locked nucleic acid
MAPK: Mitogen-activated protein kinase
MEK: MAPK kinase
miRNA: microRNA
PARP: Poly(ADP-ribose) polymerase
PI-3 K: Phosphoinositide 3-kinase
RTK: Receptor tyrosine kinase
