# Supplementary figures and images for: Targeting of HER3 with Functional Cooperative miRNAs Enhances Therapeutic Activity in HER2-Overexpressing Breast Cancer Cells
Source: Biol Proced Online. 2018 Aug 8;20:16. doi: 10.1186/s12575-018-0081-x (PMC6081814; doi:10.1186/s12575-018-0081-x)

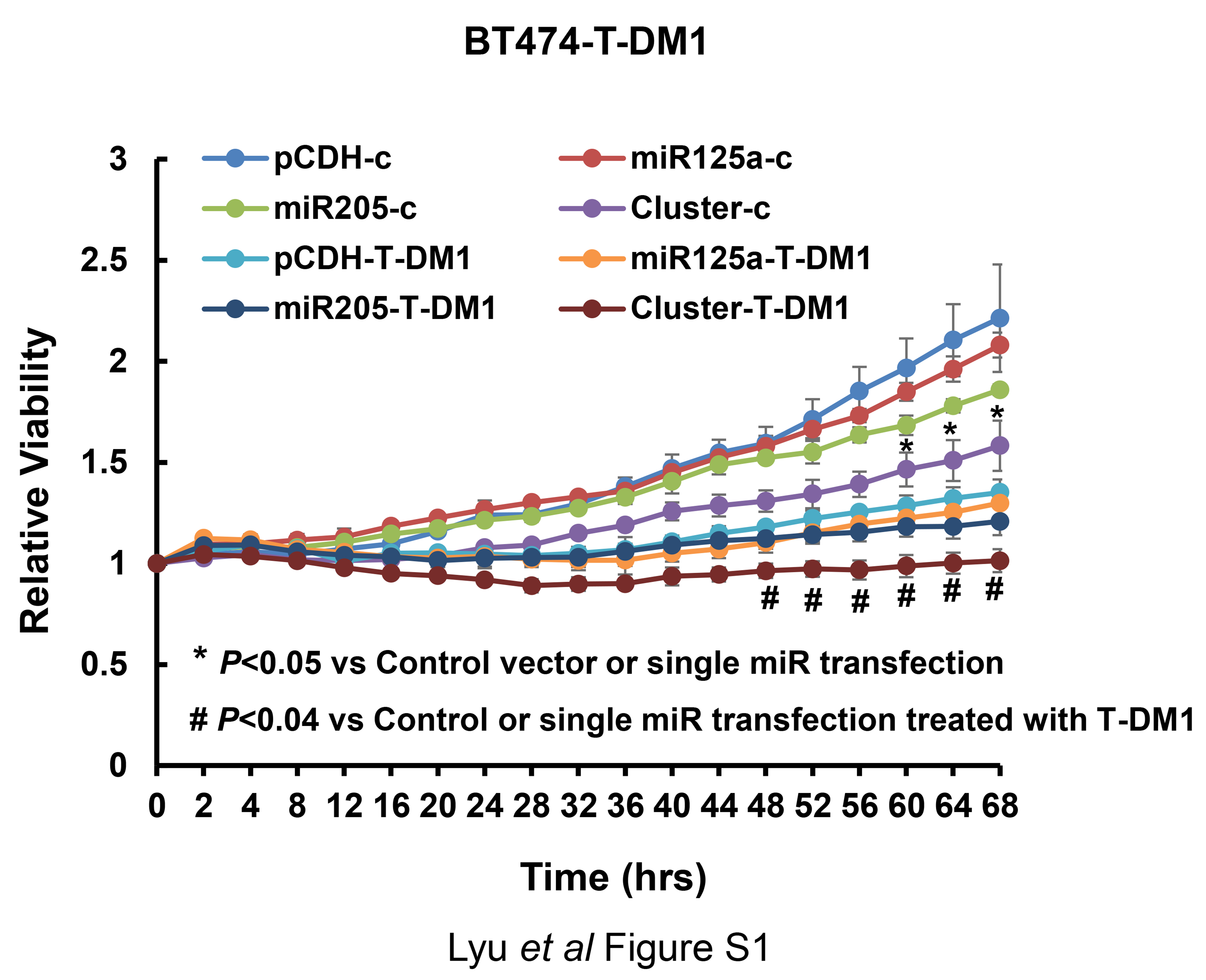

Supplement: Supplementary file 1 — Figure S1. Transfection with the multi-miRNA (cluster) lentiviral vector significantly enhanced T-DM1-induced growth inhibitory effects on BT474 cells. The stable clones of BT474 cells transfected with empty vector (pCDH), pCDH-miR-125a, or pCDH-miR-205 alone or pCDH-miR-125a-miR-205 (cluster) were seeded onto 96-well plates. After 24 hrs, the 96-well plates were then placed into the IncuCyte system to measure cell proliferation in a real time. Data show growth curves of the clones in comparison with their responses to the treatment of T-DM1 (10 μg/ml) in the indicated time period. (TIF 2615 kb) [file 12575_2018_81_MOESM1_ESM.tif]
